# Orthodontically guided bone transport in the treatment of alveolar cleft: A case report

**DOI:** 10.4317/jced.52753

**Published:** 2016-02-01

**Authors:** Estefanía Alonso-Rodríguez, Elena Gómez, Marta Otero, Rosario Berraquero, Begona Wucherpfennig, Juan Hernández-Godoy, Jorge Guiñales, Germán Vincent, Miguel Burgueño

**Affiliations:** 1 MD, Servicio Cirugía Oral y Maxilofacial Hospital Universitario La Paz. Paseo la Castellana 261, Madrid; 2 MD, PhD, Servicio Cirugía Oral y Maxilofacial Hospital Universitario La Paz. Paseo la Castellana 261, Madrid; 3 DMD, Ortodoncia Mirasierra. C/Moralzarzal 80, Madrid; 4 CDT, Vincentdental. Laboratorio Maxilofacial. C/La Palma, 74, Madrid

## Abstract

**Introduction:**

Conventional treatments are sometimes not possible in certain alveolar cleft cases due to the severity of the gap which separates the fragments. Various management strategies have been proposed, including sequential surgical interventions or delaying treatment until adulthood to then carry out maxillary osteotomies. A further alternative approach has also been proposed, involving the application of bone transport techniques to mobilise the osseous fragments and thereby reduce the gap between lateral fragments and the premaxilla.

**Case Report:**

We introduce the case of a 10-year-old patient who presented with a bilateral alveolar cleft and a severe gap. Stable occlusion between the premaxilla and the mandible was achieved following orthodontic treatment, making it inadvisable to perform a retrusive osteotomy of the premaxilla in order to close the alveolar clefts. Faced with this situation, it was decided we would employ a bone transport technique under orthodontic guidance using a dental splint. This would enable an osseous disc to be displaced towards the medial area and reduce the interfragmentary distance. During a second surgical intervention, closure of the soft tissues was performed and the gap was filled in using autogenous bone.

**Conclusions:**

The use of bone transport techniques in selected cases allows closure of the osseous defect, whilst also preserving soft tissues and reducing the amount of bone autograft required. In our case, we were able to respect the position of the premaxilla and, at the same time, generate new tissues at both an alveolar bone and soft tissue level with results which have remained stable over the course of time.

** Key words:**Alveolar cleft, bone transport, graft.

## Introduction

Cleft lip and palate are the most common congenital malformations of the head and neck. Alveolar clefts are one of the most frequent sequelae affecting these patients. There is a group of patients, primarily affected by a bilateral alveolar cleft, in which conventional treatments are not posible due to the severity of the gap which separates the fragments, alongside a paucity of soft tissue to cover the bone graft.

The main objective in the treatment of alveolar cleft is to restore a normal architecture through the osseous continuity of maxillary segments. This allows eruption of the permanent teeth adjacent to the cleft, the possibility of oral implant-supported prosthetic rehabilitation, closure of any residual oronasal fistulae and an improved aesthetic outcome through the support and elevation of the alar base.

The closure of an alveolar cleft using autogenous bone grafting was first reported in 1901 by von Eiselberg. It was much later on, in 1970, when Boyne used a bone graft obtained from the iliac crest.

Bone grafts are classified as primary or secondary depending on the age of the patient at the time of surgery. Primary bone grafting is that which is performed before the age of two. This procedure, however, has largely fallen into disuse as it leads to an impaired development of the middle third of the face and does not negate the need for subsequent re-grafting. Secondary bone grafting, in turn, can be classified as early (ages 2 to 5, primary dentition), conventional (ages 5 to 12, mixed dentition) or late (from adolescence onwards, permanent dentition). Early grafting, much like primary grafting, can also compromise subsequent facial development. Conventional grafting facilitates lateral incisor and permanent canine eruption without hampering development. Late grafting, carried out once maxillary growth has stopped, is more frequently used prior to secondary procedures such as orthognathic surgery or implant rehabilitation.

Although the most appropriate time to perform alveolar grafting has previously been a controverted subject, conventional secon-dary bone grafting utilising cancellous bone of the iliac crest prior to canine eruption is now widely regarded as being the standard treatment for alveolar cleft.

Bone grafting and gingivoperiosteoplasty, however, may be insufficient in the presence of a very wide cleft, such as in the case of a bilateral alveolar cleft, due to the difficulty in closing the gap with gingival tissue alone. One alternative is to carry out a staged closure, whilst bone transport has also been proposed as a further option.

We present the case report of a patient with a severe bilateral cleft lip, alveolus and palate, who was treated with a novel technique of alveolar bone transportation under orthodontic guidance.

## Case Report

**Figure 1:**
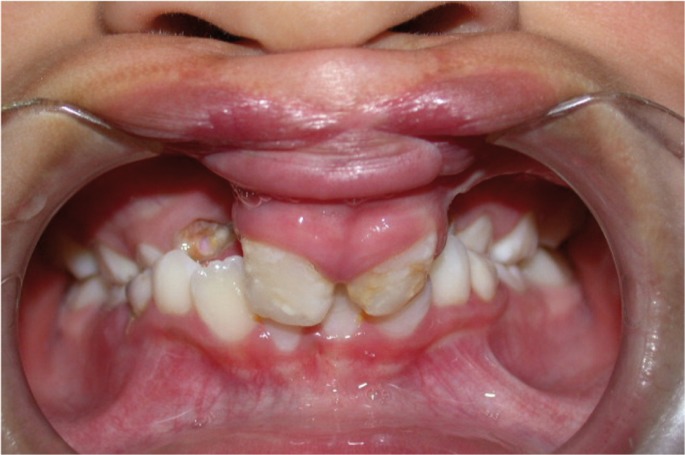
Clinical image prior to bone transport. A significant bilateral alverolar cleft can be observed. There is a transverse maxillary deficit with lateral segment collapse. The premaxilla is well positioned.

Male patient with a past medical history of severe cleft lip and palate. At 8 months, a cheilorhinoplasty was carried out in Ecuador and at the age of two he underwent a palatoplasty in our Department of Oral and Maxillofacial Surgery at Hospital Universitario La Paz, Madrid. By the age of 10 he presented an important gap (8mm) with a bilateral alveolar cleft, in addition to an anterior palatal fistula. Despite a well positioned premaxilla, there was an associated transverse maxillary deficit with collapse of the lateral segments. The patient was found to have class I dental relationship anteriorly and a bilateral crossbite posteriorly. His permanent canines had not yet erupted (Fig. 1).

**Figure 2:**
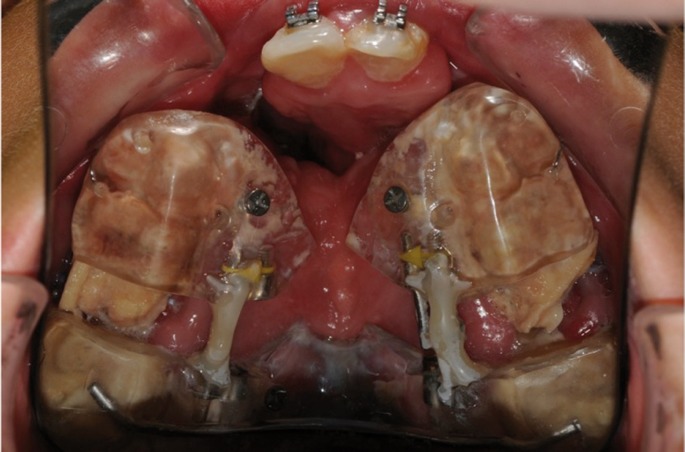
Patient-specific design of the bone transport device in the mouth of the patient.

Pre-surgical orthodontic treatment to position the premaxilla and expand the lateral segments using a Hyrax expander device was followed by orthodontically-guided bone transport to close the alveolar cleft (Fig. 2).

Once informed consent was accepted, using an intraoral approach under general anaesthesia with nasotracheal intubation, we carried out interdental osteotomies in lateral sectors 15-16 and 25-26. Horizontal osteotomies were also performed in each sector using an oscillating saw, approximately 5mm from the dental roots, in order to avoid damaging the definitive canines which had yet to erupt. The two transport discs were designed in this way, each being attached to two dental pieces and forming the mobile part of the transport system. The transport device was fitted once the osteotomies had been carried out.

After a latency period of 5 days, the transporter was activated via wheels similar to those used for palatine expansion and advanced at a rate of 0.25mm / 12 hours. Advancement continued until bony contact between the tooth-bearing segments and the premaxilla was achieved. Following a 10-week latency period, once informed consent was accepted, a second surgery under general anaesthesia was performed. During this intervention the transporter was removed and an alveoloplasty with interposed cancellous bone chips from the iliac crest was used to close the alveolar and palatine clefts.

**Figure 3:**
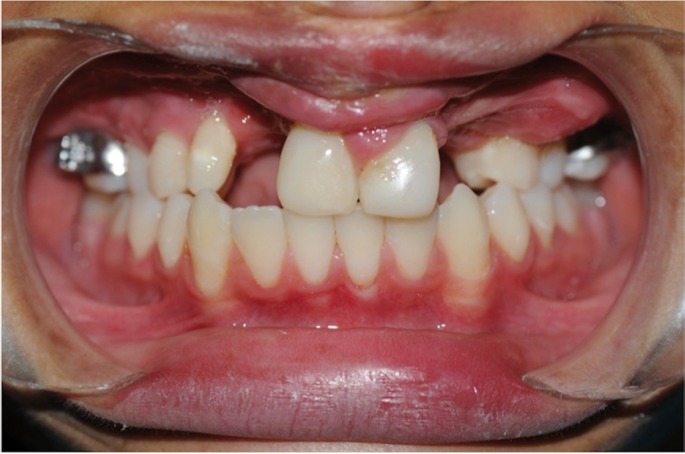
Image 4 years after bone transport. Maxillary arch continuity can be observed, together with a good alveolar ridge height.

At 5 years of follow-up we obseve continuity of the maxillary arch and a good alveolar ridge height (Fig. 3). No complications of note arose during the transport and consolidation stages. However, at present some problems exist regarding the eruption of the definitive canines, which are being displaced from their usual position relative to the premolars due to advancement of these pieces. This disruption is being managed orthodontically and it is anticipated that dental crowns will be used to camouflage it.

## Discussion

Conventional secondary bone grafting, just prior to canine eruption, is widely accepted as the standard treatment for alveolar clefts. However, in the presence of a very wide cleft, a deficit of soft tissue will limit its closure and this, in turn, is associated with a high risk of treatment failure. Bone transport is an effective alternative as it helps create new alveolar bone and new gingiva between the alveolar segments. The gap can be significantly reduced to such an extent that, even in the absence of complete contact between the segments, the remaining distance is sufficient to allow closure of the cleft with a conventional bone graft at a later date by carrying out an alveoloplasty.

Bone transport can be tooth-supported, bone-supported or a hybrid of the two. Bone-supported transporters follow a more linear direction. The main advantage of tooth-supported transport is that the segment is transported following the direction of the dental arch, generating a maxillary bone with a normal curvature. Some studies have observed that in dentally-supported transporters the dental movement is greater than that of the bone (1,2). However, studies such as that of Liou *et al.* (3) observed the opposite. In the latter study they carried out interdental distraction on 11 patients, obtaining an osseous movement of 10-20mm, whilst observing only 1mm of dental movement. There may be some unpredictable rotation of teeth in the distracted segments but, once distraction ends, orthodontic treatment continues and the teeth can move more easily in the yet immature distracted bone (3). Another advantage of tooth-supported transporters is that these can be removed directly without the need for a further surgical intervention. In our case, however, the bone transport devices were removed under general anaesthesia at the time of closing the palatine cleft and carrying out an alveoloplasty during the second surgical intervention.

In the presence of a very wide cleft, it is often difficult to close the gap due to a lack of sufficient soft tissue. We feel that orthodontically-guided bone transportation is a good option for creating new alveolar space with mixed osseous and gingival content, some distance from the cleft and which allows closure of the alveolar gap. As we can see in the case here presented, we were able to achieve 8mm of new bone on each side through the distraction process. In the orthopantomography we could observe the advancement of the bone transport disc and the results at five years of follow up continue to remain stable.

Liou recommends carrying out this technique in clefts that are wider than a canine. Fukuda *et al.* (4) highlight that there is a high risk of failure when grafting clefts over 11mm. Koh *et al.* (5) also take into account the position of the premaxilla and recommend the following treatment protocol for biltateral alveolar clefts: in small clefts (< 8 mm) a bone graft is sufficient; in wide clefts (> 8 mm) with a favourable premaxillary position, they recommend reducing the gap using interdental bone distraction and grafting afterwards if necessary; in wide clefts with an unfavourable premaxillary position, they recommend repositioning the premaxilla and, depending upon the size of the resultant gap, distraction and / or grafting.

The surgical technique itself is a simple one and carries with it a very low complication rate. It is important to create a customized transport device based on dental molds and to have a very close working relationship with the orthodontist. Some authors recommend that the distracted segments contain at least two dental pieces to ensure an adequate blood supply. Orthodontic treatment is important both before and after bone transport. The lateral segments in bilateral alveolar fissures tend to be collapsed, resulting in severe transverse deficits. It is important to expand the maxillary arch (with devices such as the quad helix, reverse quad helix, hyrax, etc) even if the gap increases as the expansion is carried out, and to align the dental pieces. After transport, teeth from posterior sectors will have been displaced towards more anterior sectors. Subsequent orthodontic treatment should be aimed at maintaining the expansion and bringing treatments to a close.

There are various distraction protocols which describe latency periods ranging from three (6) to six days (7); different distraction rates which range from daily 0.5mm increments (6-8) to weekly reduction rates (3); varying consolidation periods which go from one day (3) up to 10 weeks (7). We favoured a 5 day latency based on wound healing, a rate of 0.25mm twice daily and a consolidation period of 10 weeks. The orthodontic treatment which followed the distraction was started as soon as soft tissue closure was complete and there was good evidence of primary wound healing.

Using this technique, it has been possible to create maxillary space with good results, remaining stable over time and which have not affected velopharyngeal function (3,9).

## Conclusions

Orthodontically guided bone transport is an effective technique for reducing the gap present in severe alveolar clefts and produces stable results over time. It allows for the effective formation of bone and soft tissues which follow the shape of the maxillary arch. It is an easy technique with multiple advantages and a high success rate.
